# The Relationship between Brain Morphology and Polysomnography in Healthy Good Sleepers

**DOI:** 10.1371/journal.pone.0109336

**Published:** 2014-10-02

**Authors:** Matthias A. Reinhard, Wolfram Regen, Chiara Baglioni, Christoph Nissen, Bernd Feige, Jürgen Hennig, Dieter Riemann, Kai Spiegelhalder

**Affiliations:** 1 Department of Psychiatry and Psychotherapy, University Medical Center Freiburg, Freiburg, Germany; 2 Department of Diagnostic Radiology, University Medical Center Freiburg, Freiburg, Germany; Centre Hospitalier Universitaire Vaudois Lausanne - CHUV, UNIL, Switzerland

## Abstract

**Background:**

Normal sleep continuity and architecture show remarkable inter-individual variability. Previous studies suggest that brain morphology may explain inter-individual differences in sleep variables.

**Method:**

Thirty-eight healthy subjects spent two consecutive nights at the sleep laboratory with polysomnographic monitoring. Furthermore, high-resolution T1-weighted MRI datasets were acquired in all participants. EEG sleep recordings were analyzed using standard sleep staging criteria and power spectral analysis. Using the FreeSurfer software for automated segmentation, 174 variables were determined representing the volume and thickness of cortical segments and the volume of subcortical brain areas. Regression analyses were performed to examine the relationship with polysomnographic and spectral EEG power variables.

**Results:**

The analysis did not provide any support for the a-priori formulated hypotheses of an association between brain morphology and polysomnographic variables. Exploratory analyses revealed that the thickness of the left caudal anterior cingulate cortex was positively associated with EEG beta2 power (24–32 Hz) during REM sleep. The volume of the left postcentral gyrus was positively associated with periodic leg movements during sleep (PLMS).

**Conclusions:**

The function of the anterior cingulate cortex as well as EEG beta power during REM sleep have been related to dreaming and sleep-related memory consolidation, which may explain the observed correlation. Increased volumes of the postcentral gyrus may be the result of increased sensory input associated with PLMS. However, due to the exploratory nature of the corresponding analyses, these results have to be replicated before drawing firm conclusions.

## Introduction

Normal sleep shows remarkable variability among individuals. This variability applies to polysomnographically determined sleep variables like sleep duration and sleep efficiency [1], sleep spindles [2], EEG sigma activity [3] and total EEG spectral power [4]. In contrast, when analyzing night-to-night variability within an individual, some sleep variables are remarkably stable [5]. This stability was found in standard polysomnographic variables [1], and especially in EEG spectral power values during different sleep stages [1], [4] as well as in the frequency and topography of sleep spindles [2]. De Gennaro et al. used the term “fingerprint” to describe this high intra-individual stability in sleep EEG parameters [3]. It is plausible to assume that this high intra-individual stability results from stable neurobiological factors, as for example the morphology of the brain which is closely linked to brain function [6].

Recently, some research has focused on elucidating the association between brain morphology and inter-individual differences in sleep parameters [5], [7]. Up to now, these studies focused on EEG spectral power variables because of their particularly high intra-individual stability. Buchmann et al. found a significant correlation between the size of the anterior corpus callosum and the maximal spectral power of slow wave activity (SWA) [7]. Saletin et al., instead, reported that the slow wave amplitude correlated positively with the gray matter volume of the orbitofrontal cortex and the cingulate cortex [5]. Only one study to date has investigated the correlation between subjectively reported sleep continuity variables and specific brain structures in healthy individuals [8]. The authors found a positive correlation between hippocampal volumes in children and self-reported sleep duration.

In summary, no clear picture has emerged from previous investigations and none of the reported findings has been replicated. Furthermore, the association between standard polysomnographic parameters and brain morphology have not been investigated up to now. Therefore, the aim of the current study was to further contribute to our understanding of the relationship between brain morphology and polysomnographic variables by investigating a well-defined sample of healthy good sleepers. The analyses were carried out in two steps. First, the following a-priori hypotheses were tested based on the results of previous work: (1) SWA is significantly correlated with the morphology of the orbitofrontal cortex, the cingulate cortex and the anterior corpus callosum; (2) There is a significant positive correlation between the hippocampal volume and total sleep time. Second, an explorative approach was chosen in order to further explore the associations between polysomnographic variables and other variables of brain morphology.

## Materials and Methods

### Participants

Forty healthy subjects, who were recruited through local advertisements, were included in the current study. Two participants were excluded from the analysis because of pathologic MRI scans. Thus, the final sample consisted of 38 healthy good sleepers. The data stems from the control group of a project on insomnia morphometry [9], [10]. A semistandardized psychiatric and sleep-related interview was conducted by an experienced psychiatrist to rule out any history of psychiatric disorder, shift work, or sleep disorder. Furthermore, all participants underwent a standard physical examination, including electrocardiogram, electroencephalogram (EEG), and routine laboratory investigation (blood cell count; liver, renal and thyroid function) to exclude those with serious medical conditions. All participants were right-handed, as assessed with the Edinburgh Handedness Inventory [11], and free of any psychoactive medication. Participants with a periodic leg movements (PLMS) during sleep arousal index per total sleep time (TST) of more than 5.0/h or a sleep apnea index per TST of more than 5.0/h were not included in the current study. The study was conducted in accordance with the Declaration of Helsinki. The study protocol was approved by the Institutional Review Board of the University Medical Center Freiburg. All participants gave their informed written consent prior to inclusion in the study.

### Polysomnography

All participants underwent 2 consecutive nights of polysomnography. The first night served as an adaptation and screening night to rule out sleep apnea, periodic leg movements in sleep, and occult sleep disorder pathology. Sleep was recorded on 24-channel Sagura EEG-polysomnographs for 8 h from “lights out” (22:00 to 23:00) until “lights on” (06:00 to 07:00). All recordings included EEG (F4-M1; C4-M1; O2-M1), electrooculogram (horizontal and vertical) and electromyogram (submental), and were scored visually by experienced raters according to the American Academy of Sleep Medicine criteria [12]. In the first night, all participants were screened for apneas and periodic leg movements by monitoring abdominal and thoracic effort, nasal airflow, oxymetry, and bilateral tibialis anterior EMG. Sleep recordings were evaluated for the following parameters of sleep continuity: TST; sleep efficiency (ratio of TST to time in bed×100%); sleep onset latency defined as time from lights out until sleep onset (defined as first epoch of stage 2); wake after sleep onset (WASO) defined as difference between sleep period time (SPT; time from sleep onset until final awakening) and TST; number of awakenings; and arousal index. Sleep architecture parameters were amounts of stages 1 and 2, slow wave sleep (SWS) and rapid eye movement sleep (REM) as percentage of SPT. Leg movements and sleep apnea were evaluated according to standard criteria. All participants had to refrain from alcohol, caffeine, and daytime naps during the recording days.

### Spectral analysis

A standard procedure was used for EEG spectral analysis (see e.g. [13]). During the night, continuous EEG (C3 referenced to the right ear) was amplified with a time constant of 0.3 s and a low pass at 70 Hz (12 dB/octave), digitized at 200 Hz and stored for off-line analysis. An all-night spectral analysis was performed on the same 30-s epochs for which sleep stages had been determined. Within each epoch, spectral power was calculated using the fast Fourier transform (FFT) algorithm from 22 windows (512-points each) overlapping by half, resulting in a spectral resolution of 0.39 Hz. Within each FFT window, the EEG was demeaned and detrended by subtracting the linear least-squares regression line before applying a Welch window and calculating the FFT. The 22 spectral power estimates were averaged to increase the stability of the estimate. The goal of the further analysis was to minimize the effects of confounding variables on the spectra averaged across epochs, such as the number of movements or arousals and other sleep parameters that can be analyzed separately. This was done by two techniques: (a) arousals and myoclonias were visually marked during staging and epochs including any such events were excluded from the analysis; (b) a fully automatic exclusion of ‘deviant’ epochs from the average was performed. Deviant epochs were those containing movements or arousals as determined during staging; furthermore, the total (0.8–48 Hz) and gamma-band (32–48 Hz) log power of each epoch were related to the corresponding median-filtered value (the median of values in the 5 min preceding and 5 min following the epoch) and an epoch was excluded if the deviation was larger than the difference between the median and the first quartile of all median-filtered values across the night. In this way, artifacts mainly restricted to low frequencies (such as EOG events) as well as those occurring mainly in higher frequencies (such as EMG contamination) were eliminated in a data-driven way. All-night spectral power averages were obtained across all artifact-free epochs of sleep stage 2 and REM sleep separately. The analysis of NREM sleep was restricted to stage 2 sleep in order to eliminate the influence of different NREM sleep stage distributions across subjects.

The logarithmic (base e) spectra for artifact-free sleep epochs were averaged across each night separately for NREM stage 2 and REM sleep. Logarithmic spectral band power was calculated after adding the spectral power values of FFT bins with center frequency within the following frequency bands: delta1 (0.1–1.0 Hz), delta2 (1.0–3.5 Hz), theta (3.5–8 Hz), alpha (8–12 Hz), sigma1 (12–14 Hz), sigma2 (14–16 Hz), beta1 (16–24 Hz), beta2 (24–32 Hz), and gamma (32–48 Hz). Additionally, the total band (0.1–48 Hz) was calculated.

### MRI acquisition and analysis

High-resolution T1-weighted MRI datasets were acquired on a 3-Tesla scanner (Magnetom TIM-Trio, Siemens, Erlangen, Germany) using an MPRAGE sequence (repetition time (TR) 2.2 sec; echo time (TE) 2.6 msec; 160 sagittal slices of 256×256 voxels, 1.0×1.0×1.0 mm^3^ [14]). The MRI investigations were carried out between 7 days and 3 months after the PSG recordings. All scans were inspected for motion artefacts and for the absence of pathologic findings by a neurologist under the supervision of a board-certified neuroradiologist.

### FreeSurfer-based morphometry

Cortical surface reconstruction and volumetric segmentation was performed using the FreeSurfer software, version 5.1.0 (Athinoula A. Martinos Center for Biomedical Imaging; http://surfer.nmr.mgh.harvard.edu/). The technical details of this procedure have been described in previous publications [15], [16], [17].

For quality control, segmentations were visually inspected for each participant by two independent raters (K.S. and W.R.) on a slice-by-slice basis. However, no manual corrections were necessary for the automatic segmentation results. 38 subcortical volumes (“aseg.stats” files) as well as cortical volume and thickness values of 68 structures (“aparc.stats” files) based on the Killiany/Desikan cortical parcellation [18] were extracted from FreeSurfer, resulting in a total of 174 measures per participant. Additionally, intracranial volume (ICV) was extracted.

### Statistical analysis

The data analysis was conducted with the software IBM SPSS Statistics 20.0 (SPSS Inc., Chicago, IL, USA). For descriptive purposes, age and sex effects were investigated. Pearson’s correlation was used to determine the association between ICV and age. A t-test for independent groups was performed to test for ICV differences between sexes. Furthermore, a multivariate ANOVA with the 174 morphometric variables as dependent variables was calculated to explore possible sex differences. Mean correlations were calculated using Fisher’s Z transformation to describe the association of age with morphometric volumes and thicknesses, respectively.

In order to investigate the relationship between polysomnographic parameters and brain morphometry, univariate linear regression analyses were carried out. In each of these analyses, one polysomnographic parameter was used as dependent variable and one of the 174 morphometric variable was used as independent variable. Intracranical volume (ICV), age and sex were included as covariates in all of these analyses. Non-standardized b-values as well as T-and p-values of the analyses are reported.

For testing hypothesis 1, NREM delta1 and delta2 power of the second night were used as dependent variables and the following variables as independent variables: left medial orbitofrontal volume, right medial orbitofrontal volume, left posterior cingulate volume, right posterior cingulate volume, volume of the anterior corpus callosum. Of note, the posterior cingulate is the region defined by FreeSurfer that most closely approximates the middle cingulate cortex described by Saletin et al. [5]. To obtain a better comparison with the results of Buchmann et al. [7], the regression analyses concerning the anterior corpus callosum were recalculated without correction for ICV and after limiting the study sample to those with an age <40 years. Hypothesis 2 was tested with TST of the second night as dependent variable and the hippocampal volume of the left and right hemisphere as independent variables. Overall, 14 hypothesis-based analyses were carried out. For each of these, the level of significance was set at p<.05 (two-tailed).

The explorative data analysis was also conducted using the above described ICV-, age- and sex-adjusted regression analyses. For each of 39 polysomnographic parameter (11 sleep continuity and sleep architecture variables of the second night [see Table 1], 8 leg movement and respiratory parameters of the first night [see Table 1], and 20 power spectral analysis parameters of the second night [see Table 2]), 174 regression analyses were calculated (in sum, 6786 regression analyses). P-values were corrected using the false discovery rate (FDR [19]) separately for each dependent variable. The FDR-corrected level of significance was set at p<.05 (two-tailed) for these analyses.

**Table 1:** Polysomnographic data of the first and second night (means ± standard deviations).

	Night 1	Night 2
Total sleep time (min)	379.1±56.2	416.3±24.0
Sleep efficiency (%)	80.2±10.0	86.7±5.0
Sleep onset latency (min)	20.7±19.0	17.8±16.5
Sleep period time (min)	446.0±46.7	457.9±22.5
Number of awakenings	38.0±15.8	35.4±13.7
Wake after sleep onset (min)	66.8±40.2	41.6±16.8
Arousal index (h^−1^)	16.9±6.1	14.2±5.7
Stage 1 (% SPT)	11.3±5.2	8.7±4.5
Stage 2 (% SPT)	49.9±8.7	53.8±5.9
SWS (% SPT)	6.8±6.6	8.9±7.2
REM (% SPT)	17.1±5.1	19.5±3.8
Leg movements (during TST)	39.1±34.6	-
Leg movements with arousal (during TST)	4.9±7.7	-
PLMS (during TST)	14.4±21.9	-
PLMS with arousal (during TST)	2.3±4.8	-
PLMS index (h^−1^ TST)	2.4±3.7	-
PLMS index with arousal (h^−1^ TST)	0.4±0.8	-
Apnea hypopnea index (h^−1^ TST)	3.6±4.0	-
Sleep apnea index (h^−1^ TST)	0.4±0.7	-

SPT: sleep period time; SWS: slow wave sleep; REM: rapid eye movement sleep; PLMS: periodic leg movements during sleep; TST: total sleep time.

**Table 2:** Power of EEG frequency spectra in REM sleep and NREM stage 2 (means and standard deviations).

		REM		NREM stage 2	
		Night 1	Night 2	Night 1	Night 2
Total	(0.1–48 Hz)	4.34±0.35	4.38±0.31	5.49±0.30	5.45±0.26
Delta1	(0.1–1.0 Hz)	3.15±0.51	3.17±0.41	4.68±0.40	4.61±0.33
Delta2	(1.0–3.5 Hz)	3.08±0.34	3.14±0.35	4.24±0.25	4.24±0.25
Theta	(3.5–8.0 Hz)	2.77±0.30	2.84±0.33	3.50±0.33	3.53±0.35
Alpha	(8–12 Hz)	1.89±0.36	1.93±0.39	2.73±0.41	2.75±0.45
Sigma1	(12–14 Hz)	0.43±0.39	0.43±0.43	1.59±0.50	1.56±0.55
Sigma2	(14–16 Hz)	0.17±0.38	0.16±0.41	0.67±0.50	0.63±0.54
Beta1	(16–24 Hz)	1.02±0.36	1.02±0.39	0.83±0.34	0.79±0.38
Beta2	(24–32 Hz)	0.00±0.54	–0.02±0.60	–0.34±0.35	–0.38±0.44
Gamma	(32–48 Hz)	–1.59±0.58	–1.62±0.62	–1.69±0.47	–1.75±0.51

REM = rapid eye movement sleep; NREM = non rapid eye movement sleep.

## Results

### Sample characteristics

The group consisted of 17 men and 21 women with a mean age of 39.6±8.9 years (range: 27–57 years). The mean intracranial volume (ICV) was 1087.7±100.9 cm^3^. ICV was not correlated with age (*r* = −.11, *p* = .52), and men had a higher ICV than women (*t*[36] = 5.62, *p*<.001).

The mean correlations of age with morphometric volumes and thicknesses were *r* = −.32, (*t*[105] = −13.46, *p<*.001) and *r* = −.26 (*t*[67] = −10.52, *p*<.001), respectively. A multivariate ANOVA with all 174 morphometric variables as dependent variables missed overall significance (*F*[36]; [1] = 5.01, *p* = .34) for the independent variable sex.

Polysomnographic data of the sample are presented in Table 1. Data for power of EEG frequency spectra are presented in Table 2.

### Testing a-priori hypotheses

Hypothesis 1: Regression analyses did not reveal any significant association between NREM delta1 or delta2 power and the medial orbitofrontal volume (NREM delta1 and left medial orbitofrontal volume: *b* = 8.1*10^−5^, *t*[33] = 0.70, *p* = .49; NREM delta2 and left medial orbitofrontal volume: *b* = 1.3*10^−4^, *t*[33] = 1.70, *p* = .10; NREM delta1 and right medial orbitofrontal volume: *b* = 8.2*10^−5^, *t*[33] = 0.80, *p* = .43; NREM delta2 and right medial orbitofrontal volume: *b* = 8.0*10^−5^, *t*[33] = 1.18, *p* = .25), posterior cingulate volume (NREM delta1 and left posterior cingulate cortex: *b* = −5.1*10^−5^, *t*[33] = −0.42, *p* = .68; NREM delta2 and left posterior cingulate cortex: *b* = 1.2*10^−4^, *t*[33] = 1.61, *p* = .12; NREM delta1 and right posterior cingulate cortex: *b* = 7.4*10^−5^, *t*[33] = 0.68, *p* = .50; NREM delta2 and right posterior cingulate cortex: *b* = 6.1*10^−5^, *t*[33] = 0.85, *p* = .40), and the volume of the anterior corpus callosum (NREM delta1: *b* = 1.2*10^−4^, *t*[33] = 0.28, *p* = .78; NREM delta2: *b* = 4.1*10^−4^, *t*[33] = 1.47, *p* = .15). After limiting the study sample to those with an age <40 years (11 men, 9 women; age: 32.3±3.4 years) and eliminating ICV from the list of covariates (to obtain a better comparison with the results of Buchmann et al. [7]), regression analyses revealed no significant relationship between the volume of the anterior corpus callosum and NREM delta1 or delta2 (NREM delta1: *b* = −3.6*10^−4^, *t*[33] = −0.63, *p* = .54; NREM delta2: *b* = 2.2*10^−4^, *t*[33] = 0.64, *p* = .53).

Hypothesis 2: Hippocampal volumes were not significantly correlated with TST (left hippocampus: *b* = 4.7*10^−3^, *t*[33] = 0.38, *p* = .71; right hippocampus: *b* = 1.9*10^−3^, *t*[33] = 0.19, *p* = .85).

### Explorative analyses

Table 3 lists the results of the explorative data analyses that survived FDR correction.

**Table 3:** Significant relationships between brain structures and polysomnographic variables after FDR correction (adjusted p-values are reported).

				*b*	*t*(33)	*p*
**Gyrus postcentralis**	L	V	leg movements	0.022	4.57	.01
**Gyrus postcentralis**	L	V	PLMS	0.015	5.36	.001
**Gyrus postcentralis**	L	V	PLMS index	2.6*10^−3^	5.13	.002
**Caudal ACC**	L	T	REM beta2	2.109	4.66	.01

L = left hemisphere; V = volume; PLMS: periodic leg movements during sleep; ACC: anterior cingulate cortex; T = thickness; REM = rapid eye movement sleep.

#### 3.3.1 Standard polysomnographic variables

**Figure 1:**
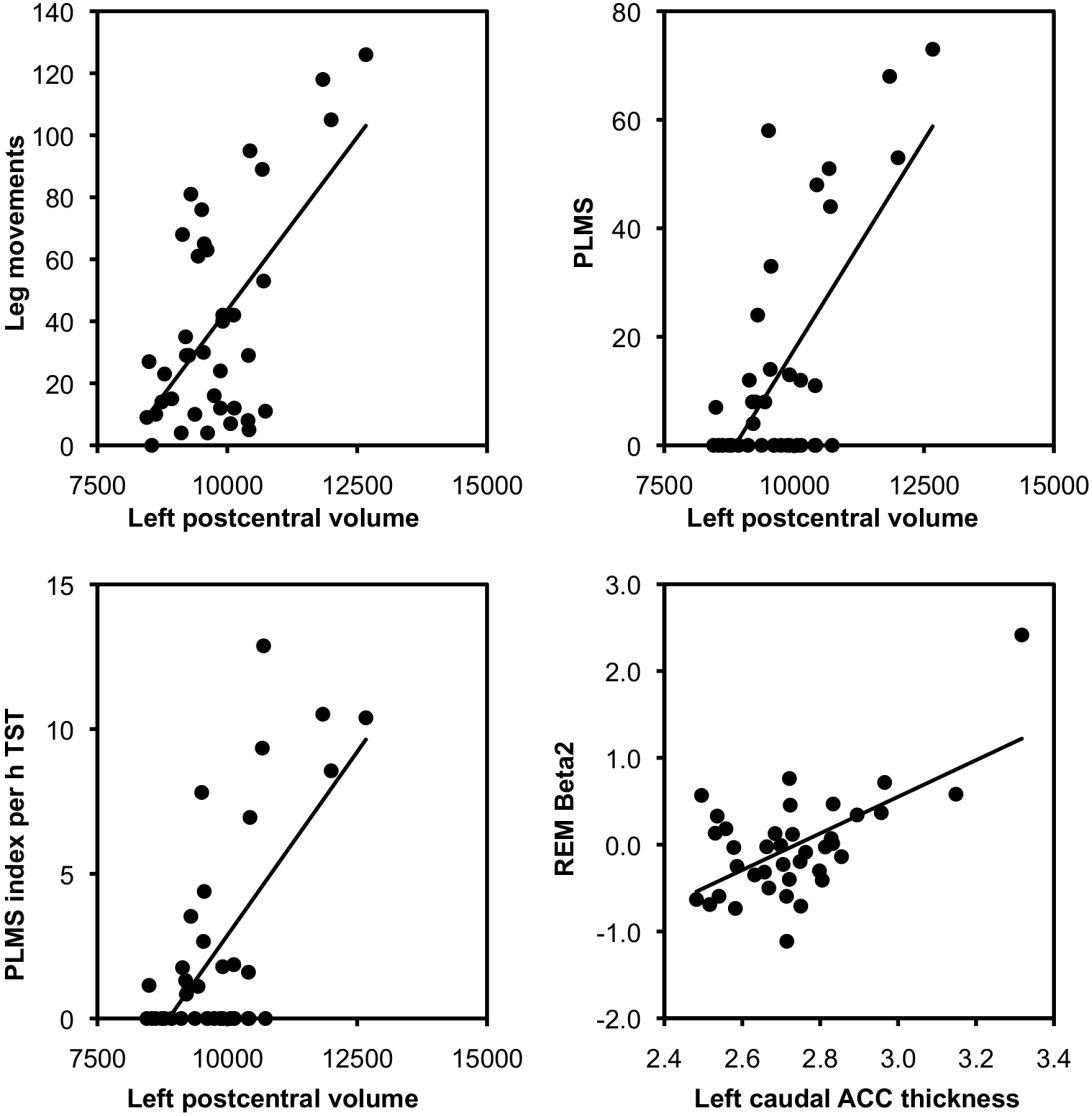
Scatter plots of relationships between brain structures and polysomnographic parameters. Presented volumes are age-, sex- and ICV-adjusted by using the residual method. Solid lines represent regression lines. ACC = anterior cingulate cortex; ICV = intracranial brain volume; PLMS = periodic leg movements during sleep; REM = rapid eye movement sleep; TST = total sleep time.

The volume of the left postcentral gyrus was positively correlated with the number of leg movements, the number of PLMS, and the PLMS index (Table 3). Figure 1 shows the corresponding scatter plots. Of note, the associations between the right-hemispheric postcentral volume and the leg movement-related variables pointed in the same direction as the ones for the left-hemispheric postcentral volume. However, these results did not survive FDR correction (number of leg movements: *b* = 0.020, *t*[33] = 3.03, *p* = .004, uncorrected, *p* = 0.38, corrected; number of PLMS: *b* = 0.013, *t*[33] = 3.30, *p* = .002, uncorrected, *p* = 0.20, corrected; PLMS index: *b* = 2.1*10^−3^, *t*[33] = 3.02, *p* = .005, uncorrected, *p* = 0.17, corrected). The correlations between the left postcentral volume and arousal-associated leg movement variables did also not survive FDR correction (leg movement with arousal: *b* = 4.1*10^−3^, *t*[33] = 3.26, *p* = .003, uncorrected, *p* = 0.45, corrected; PLMS with arousal: *b* = 2.4*10^−3^, *t*[33] = 3.14, *p* = .004, uncorrected, *p* = 0.62, corrected; PLMS arousal index: *b* = 3.9*10^−3^, *t*[33] = 3.24, *p* = .003, uncorrected, *p* = 0.47, corrected).

#### 3.3.2 Spectral analysis

There was a significant relationship between the thickness of the left caudal anterior cingulate cortex (ACC) and beta2 power in REM sleep (see Table 3). Figure 1 shows the corresponding scatter plot. One participant showed a remarkably increased thickness of the left caudal ACC and remarkably high beta2 values during REM sleep (thickness: 3.5 standard deviation [SD] above of the sample’s mean; beta2: 4.5 SD above the sample’s mean). This participant showed increased beta activity in both nights and selectively during REM sleep. However, with regard to other EEG or sociodemographic variables, no explanation for this deviation could be found. Brain segmentation of this participant was rechecked and, again, rated as adequate. The scatter plots show that this participant strongly affects the regression analyses. As a consequence, these results should be interpreted with caution.

## Discussion

### Testing a-priori hypotheses

The presented data did not provide any support for the a-priori formulated hypotheses of an association between brain morphology and polysomnographic variables. Several potential reasons for this have to be discussed.

First, the current study used an automated segmentation technique based on the FreeSurfer tool. While Buchmann et al. [7] used the same software for their analyses, Saletin et al. [5] investigated their sample by using voxel based morphometry, which may detect more subtle differences in localized areas of the brain and may be better suited for subcortical structures with small surface areas (see [18]). Furthermore, in contrast to Buchmann et al. [7], the analyses of the current study were corrected for the influence of ICV, which is a standard technique in brain morphometry studies to reduce the impact of different brain sizes.

Second, the EEG data was processed differently. Saletin et al. [5] calculated SWA amplitude for selected NREM sleep episodes (including stage 2 and SWS) in the frequency range 0.5–4 Hz. Buchmann et al. [7], in comparison, used a rather unique parameter, the maximal SWA (0.5–4.5 Hz) during NREM sleep. The current study instead used all-night power spectral analysis within defined frequency bands, a common method in this field [20].

Last, with respect to the non-replication of the association between total sleep time and hippocampus volumes, the current study investigated adults in contrast to the investigation by Taki et al. [8] who investigated children aged 5 through 18 years. It has also to be noted that Taki et al. had a considerably larger power due to a larger sample size (*N* = 290). Finally, the results are difficult to compare because Taki et al. investigated the association between brain morphology and subjective sleep (measured with a self-assessment questionnaire) without including polysomnographic measurements.

Despite these differences between this study and previous investigations, the current results suggest that other variables than brain morphology contribute substantially to the explanation of variance in PSG parameters.

### Explorative analyses

The results of the current study suggest a positive correlation between the volume of the left postcentral gyrus and leg movements during sleep. The association between the volume of the right postcentral gyrus and leg movements during sleep pointed in the same direction. Up to now, the association between brain morphology and leg movements during sleep has not been studied. However, PLMS are a frequent symptome of the restless legs syndrome (RLS) [21], a condition that has been suggested to be associated with morphometric postcentral gyrus alterations before (see [22] for an overview). Furthermore, Margariti et al. [23] showed an increased activation of the left postcentral gyrus during nocturnal leg movements in RLS patients using fMRI. As morphometric studies suggest a positive impact of the activity of a structure on its size [6], an increased postcentral gyrus volume may be the consequence of an increased activity of the postcentral gyrus, an observation that fits well with the uncomfortable and unpleasant sensations of RLS patients. The current study suggests that even PLMS without RLS are associated with an increased postcentral volume, maybe due to a similar mechanistic pathway.

A further important observation of the current study was the positive association between the thickness of the left caudal ACC and EEG beta2 power during REM sleep. In general, beta power is assumed to be a marker of local neuronal processing [24]. However, the underlying causes and/or consequences of beta power during REM sleep remain unclear. High-frequency activity during REM sleep may represent dreaming [25], memory consolidation [26], [27] or, more broadly, a chronic increase of arousal levels [28]. Interestingly, the ACC, as an important interface for the regulation and integration of cognitive and emotional processes [29] has also been linked to dreaming and memory processes [30], [31]. With respect to the link between both ACC and EEG beta power to arousal levels, it should be noted that a recent morphometric investigation found increased ACC volumes in primary insomnia patients [32], a disorder which is characterized by increased arousal levels [33], which are, in turn, assumed to be reflected in increased levels of nocturnal spectral beta power [28]. This is in line with the results of the current investigation and suggests that the ACC may be involved in the modulation of arousal levels during sleep. However, it has to be noted that the causal pathways between ACC thickness, spectral beta power during REM sleep and the above-mentioned sleep-related processes (dreaming, sleep-related memory consolidation, arousal) remain unclear due to the cross-sectional design of the current investigation.

### Limitations

Several limitations have to be discussed with respect to the current results and interpretations. First, although the current sample is well characterized, it is a convenience sample possibly reducing external validity. In particular, the sample had a comparably low sleep efficiency and high number of awakenings [34]. However, in comparison with previous studies the present sample appears to be representative of other healthy participants of the sleep laboratory in Freiburg [35]. Second, it should be noted that, in the current study, PLMS was measured only during the first night. As Hornyak et al. reported a large variability in the occurrence of PLMS [36] future studies may investigate the association between brain morphology and PLMS measured across multiple nights. Third, subcortical structures with smaller surface areas are probably less reliably segmented by FreeSurfer than cortical structures [18], and, thus, voxel-based morphometry may be a better option for subcortical structures. Last, the current study used a cross-sectional design. A longitudinal approach would reveal maturation processes and neural plasticity. In this context, a study of a sample of children and adolescents would be of particular interest.

### Conclusions

The presented data did not provide any support for the a-priori formulated hypotheses regarding the association between brain morphology and polysomnographic variables. However, exploratory data analysis revealed a significant relationship between the postcentral volume and the occurrence of PLMS which may be the result of an increased sensory input associated with PLMS. Additionally, the ACC thickness was positively associated with beta2 power during REM sleep. This may be explained by an involvement of both ACC and beta power in dreaming, sleep-related memory consolidation and arousal levels.
