# Correction: The Relationship between Brain Morphology and Polysomnography in Healthy Good Sleepers

**DOI:** 10.1371/journal.pone.0116448

**Published:** 2014-12-26

**Authors:** The PLOS ONE Staff

The following information is missing from the Funding section: The article processing charge was funded by the open access publication fund of the Albert Ludwigs University Freiburg.
